# Effect of Dorsal and Ventral Hippocampal Lesions on Contextual Fear Conditioning and Unconditioned Defensive Behavior Induced by Electrical Stimulation of the Dorsal Periaqueductal Gray

**DOI:** 10.1371/journal.pone.0083342

**Published:** 2014-01-03

**Authors:** Carolina Irurita Ballesteros, Bruno de Oliveira Galvão, Silvia Maisonette, J. Landeira-Fernandez

**Affiliations:** Departamento de Psicologia, Pontifícia Universidade Católica do Rio de Janeiro, Rio de Janeiro, RJ, Brazil; McLean Hospital/Harvard Medical School, United States of America

## Abstract

The dorsal (DH) and ventral (VH) subregions of the hippocampus are involved in contextual fear conditioning. However, it is still unknown whether these two brain areas also play a role in defensive behavior induced by electrical stimulation of the dorsal periaqueductal gray (dPAG). In the present study, rats were implanted with electrodes into the dPAG to determine freezing and escape response thresholds after sham or bilateral electrolytic lesions of the DH or VH. The duration of freezing behavior that outlasted electrical stimulation of the dPAG was also measured. The next day, these animals were subjected to contextual fear conditioning using footshock as an unconditioned stimulus. Electrolytic lesions of the DH and VH impaired contextual fear conditioning. Only VH lesions disrupted conditioned freezing immediately after footshock and increased the thresholds of aversive freezing and escape responses to dPAG electrical stimulation. Neither DH nor VH lesions disrupted post-dPAG stimulation freezing. These results indicate that the VH but not DH plays an important role in aversively defensive behavior induced by dPAG electrical stimulation. Interpretations of these findings should be made with caution because of the fact that a non-fiber-sparing lesion method was employed.

## Introduction

Defensive behavior has been widely modeled in animals to investigate the neurobiology of anxiety disorders. Contextual fear conditioning is one of the simplest and most rapid behavioral paradigms for studying learned aspects of defensive behavior. In this paradigm, a rat is placed in a novel chamber. A few minutes later, a brief, unsignaled footshock is administered, and the rat is removed from the chamber. When the rat is later returned to the same chamber in the absence of the aversive stimulus (i.e., the next day), it presents a typical freezing response (i.e., thigmotaxis) usually next to an object, such as a corner or wall. Such a freezing response is not observed when the animal is placed in a new chamber not associated with footshock, indicating that this defensive behavior is a conditioned response to contextual cues previously associated the aversive unconditioned stimulus [1].

Electrical stimulation of the dorsal periaqueductal gray matter (dPAG) in rats also generates a clear set of defensive responses. A gradual increase in electrical stimulation of the dPAG, at lower intensities, produces a defensive freezing posture accompanied by piloerection and exophthalmos. As stimulation continues, vigorous escape responses, such as jumping and running, appear at higher intensities [2]. After the cessation of dPAG electrical stimulation at the escape threshold, a freezing response, termed post-dPAG stimulation freezing, is observed [3]. This freezing response has been shown to remain at a high level when the animal is placed in a new context immediately after the interruption of the electrical stimulation of the dPAG at the escape threshold [4]. Therefore, post-dPAG stimulation freezing can be considered an unconditioned response to dPAG stimulation [5].

Different hippocampal subregions have been established to play pivotal roles in conditioned and unconditioned defensive behavior that animals display in response to threatening situations. Numerous studies have shown that lesions of the dorsal hippocampus (DH) disrupted the acquisition of conditioned freezing in response to contextual cues but not in response to a discrete cue, such as a tone or light [6]–[10]. Several pharmacological studies have indicated that the DH might regulate unconditioned defensive responses to threatening situations, such as in the elevated plus maze [11]–[14], open field [11], social interaction test [15], [16], defensive burying test [12], and Vogel conflict test [14].

However, lesion data suggest that destruction of the of the ventral hippocampus (VH) also decreases unconditioned defensive behaviors, such as in the elevated plus maze [17], [18], elevated T-maze [19], open field [17], defensive burying test [16], light/dark transition test [20], social interaction test [18], and unconditioned freezing in response to cat odor [21]. Furthermore, previous studies have reported that lesions of the VH led to deficits in conditioned freezing in response to discrete and contextual cues [9], [10], [22].

These findings suggest that the DH and VH may differentially participate in aversively motivated responses. Importantly, the role of the DH and VH in defensive behaviors induced by dPAG electrical stimulation is unknown. To address this issue, the present study measured electrical thresholds of defensive freezing and escape behaviors triggered by dPAG stimulation in rats that received DH or VH electrolytic lesions. Freezing behavior was measured after the termination of electrical stimulation of the dPAG at the escape threshold. As a positive control, conditioned freezing was also assayed within the same animals subjected to a contextual fear-conditioning procedure at the end of the dPAG electrical stimulation experiment.

## Materials and Methods

### Animals

Male albino Wistar rats, born and reared in the colony room of the Psychology Department, Pontifícia Universidade Católica of Rio de Janeiro, were used as subjects. The animals were housed in groups of six in polycarbonate cages (18×31×38 cm), with food and water provided *ad libitum*. Room temperature was controlled (24±1°C), with a 12 h/12 h light/dark cycle (lights on 7:00 AM–7:00 PM). All of the experiments were conducted during the light phase of the cycle. The animals weighed 250–350 g at the beginning of the experiments. All of the experimental protocols were approved by the ethics committee of the Psychology Department at Pontifícia Universidade Católica do Rio de Janeiro, Brazil and conformed with the Brazilian Society of Neuroscience and Behavior Guidelines for Care and Use of Laboratory Animals (SBNeC), in accordance with ARRIVE guidelines [23].

### Surgery

All of the rats were anesthetized with an intraperitoneally injection of xylazine and ketamine (100 mg/kg) and placed in a stereotaxic frame (David Kopf Instruments, Tujunga, CA, USA). The upper incisor bar was set 3.3 mm below the interaural line, such that the skull was leveled between bregma and lambda. Bilateral electrolytic lesions of the DH and VH were made by passing a 5 mA anodal current for 20 s through a stainless steel insect pin (size 00) insulated with baked epoxylite except for the cut tip. A cathode clamped to the tail completed the circuit. The current was delivered by a lesion-generating device (DelVechio, Ribeirão Preto, Brazil). Based on the Paxinos and Watson (1986) rat brain atlas, the stereotaxic coordinates for DH lesions were −2.8 mm posterior to bregma, ±1.8 mm from the midline, and 3.8 mm ventral from the surface of the skull. The stereotaxic coordinates for VH lesions were −5.2 mm posterior to bregma, ±5.3 mm from the midline, and 6.0 mm ventral from the surface of the brain. Animals that were assigned to the sham lesion group underwent identical procedures, with the exception that no electrical current was delivered.

After the lesioning and sham procedures, all of the animals were implanted with a unilateral bipolar electrode aimed at the dPAG (1.9 mm lateral to lambda at a depth of 5.1 mm below the skull surface). The electrode stand was anchored to the skull by one small screw and autopolymerizing resin. The electrode was made of stainless steel wire, 160 µm diameter, insulated except at the cross-section. The electrode wire could be connected to a male pin so that it could be plugged into an amphenol socket at the end of a flexible electrical cable and used for brain stimulation.

### Apparatus

The dPAG was electrically stimulated by means of a Grass S44 square-wave stimulator (Quincy, MA, USA) connected to an oscilloscope (Tektronix, USA) that indicated the voltage drop through a 100 kΩ resistor in series with the rat. The electrodes were connected to the stimulator by means of a flexible cable and mercury swivel, which allowed free movement of the animal.

All of the experiments occurred in an observation chamber (25×20×20 cm) that was placed inside a sound-attenuating box. A red light bulb (25 W) was placed inside the box, and a video camera was mounted behind the observation chamber so that the animal's behavior could be observed on a monitor placed outside the experimental chamber. A ventilation fan attached to the chest supplied 78 dB background noise (A scale). The floor of the observation chamber consisted of 15 stainless rods (4 mm diameter) spaced 1.5 cm apart (center to center), which were wired to a shock generator and scrambler (AVS, SCR04; São Paulo, Brazil). An interface with eight channels (Insight, Riberão Preto, Brazil) connected the shock generator to a computer, which allowed the experimenter to apply an electric footshock. Ammonium hydroxide solution (5%) was used to clean the chamber before and after each subject.

### Procedure

One week after surgery, each animal was placed in the observation chamber, where it remained undisturbed for 5 min (baseline period). Brain stimulation (alternating current, 60 Hz, 15 s) was presented at 1 min intervals, with the current intensity increasing by 5 µA steps to measure the aversive thresholds. The freezing threshold was operationally defined as the lowest current intensity that produced immobility, which was defined as the total absence of movement of the body or vibrissa, with the exception of movement required for respiration. The lowest current intensity that produced running (galloping) or jumping was considered the escape threshold. After reaching the escape threshold, the electrical stimulation of the dPAG stopped, and the animal remained in the observation chamber for an additional 12 min without any stimulation. Post-dPAG stimulation freezing was scored using a time-sampling procedure. Every 2 s, the animal's freezing behavior was scored by a well-trained observer who was blind to the experimental conditions. Animals that did not reach an aversive threshold at 180 µA (peak-to-peak) were discarded from the study.

One day after the end of electrical stimulation of the dPAG, all of the animals were subjected to the contextual fear conditioning procedure. Each animal was placed in the observation chamber. Five minutes later, three unsignaled footshocks (0.6 mA) were presented at 20 s intervals. After the last footshock, the animal remained in the chamber for 2 min. The next day, the animal was returned to the observation chamber and left undisturbed for 12 min. The same time-sampling procedure was used to record freezing behavior during the 2 min period after the last footshock and the 12 min test session that occurred approximately 24 h later.

### Histology

At the end of the experiment, all of the animals were deeply anaesthetized and intracardially perfused with a 0.9% saline solution followed by a 10% formalin solution. The cannula was removed, and the brain was placed in a 10% formalin solution. Three days later, the brains were frozen, and 50 µm brain sections were cut using a cryostat and stained with Cresyl blue to localize the cannula placements and lesion locations.

A procedure adapted from Landeira-Fernandez and Grijalva [24] was used to estimate the lesion volume in the left and right hemispheres of the DH and VH. The extent of the affected brain area was evaluated with reference to the Paxinos and Watson [25] brain atlas. Histological sections were superimposed by microprojecting onto corresponding structures that represented tracings from a coronal atlas plate. Magnification was adjusted until the projected structures adjacent to the lesion corresponded to the atlas structures. Outlines of the regions that displayed apparent cell loss or chromatolysis observed in the representative section through the affected brain area were drawn to scale on tracings of the brain sections taken at appropriate anterior-posterior levels. The anterior-posterior extent of the affected brain region was estimated by calculating the division of the atlas figures that were incorporated within the lesion outline. The dorsal-ventral and medial-lateral extent of the lesion was measured within the atlas scale at the maximal lesion extensions. An estimation of the volume of the brain tissue affected was calculated using the ellipsoid formula by multiplying 4/3 π with the anterior-posterior, dorsal-ventral, and medial-lateral radius extents

### Statistical Analysis

The results are expressed as mean ± standard error of the mean (SEM). Data from the DH and VH sham-lesioned groups were analyzed using Student's *t*-test. The lesion volume was analyzed using a two-way analysis of variance (ANOVA), with lesion structure (HD and HV) and lesion hemisphere (left and right) as the between- and within-group factors, respectively. A two-way ANOVA was also used to evaluate differences in aversive thresholds. Lesioning (DH, VH, and sham) was considered the between-subjects factor, and aversive threshold (freezing and escape) was considered the within-subjects factor. Freezing behavior among the sham-, DH-, and VH-lesioned groups were analyzed using one-way ANOVA. Significant effects or interactions in the ANOVA were followed by Fisher's Least Significant Difference *post hoc* test for pairwise comparisons. The level of statistical significance was *p*<0.05.

## Results

**Figure 1:**
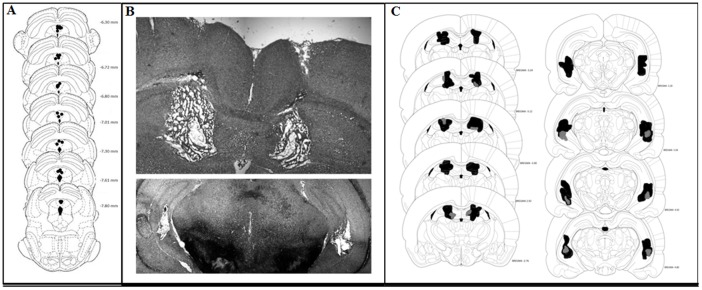
Histological results. Figure 1A. Composite of stimulation electrode tips within the dPAG. According to the Paxinos and Watson (1986) atlas, the numbers on the right-hand side of each plate indicate the distance in millimeters from bregma. Figure 1B. Histological section taken through the midpoint of a representative electrolytic lesion within the dorsal (upper) and ventral (lower) hippocampus. Figure 1C. Composite of coronal sections adapted from the Paxinos and Watson (1986) rat brain atlas. Numbers indicate the distance in millimeters from bregma. The figure shows the smallest (black) and largest (gray) damaged areas in the dorsal (left) and ventral (right) hippocampus-lesioned animals.

All of the animals included in the analysis of the present study met the criteria for electrode placement in the dPAG and bilateral electrolytic lesions of the DH and VH. The final group samples were the following: DH, *n* = 8; VH, *n* = 7; sham DH lesion, *n* = 5; sham VH lesion, *n* = 7. Fig. 1A shows the representative sites of the dPAG stimulation. Fig. 1B depicts a representative histological section of a bilateral electrolytic lesion in the DH and VH. Fig. 1C presents a composite of the representative areas of the smallest and largest lesions in the DH and VH. Histological examination of the brain slices indicated that the electrolytic lesions tended to be bilaterally symmetrical. Lesions included a cavity in the center of the lesion plus a region of chromatolysis that surrounded the cavity. No systematic damage to cortical areas was observed. The two-way ANOVA of lesion volume indicated no interaction between lesion structure and lesion hemisphere (*F*_1,13_ = 1.76, *p*>0.21). No main effect of lesion structure (*F*_1,13_ = 0.01, *p*>0.98) or lesion hemisphere (*F*_1,13_ = 0.02, *p*>0.89) was found. The mean volume for the left and right HD lesions was 3.15±0.12 mm^3^ and 3.04±0.07 mm^3^, respectively. The mean volume for the left and right HV lesions was 3.03±0.12 mm^3^ and 3.10±0.05 mm^3^, respectively.

**Figure 2:**
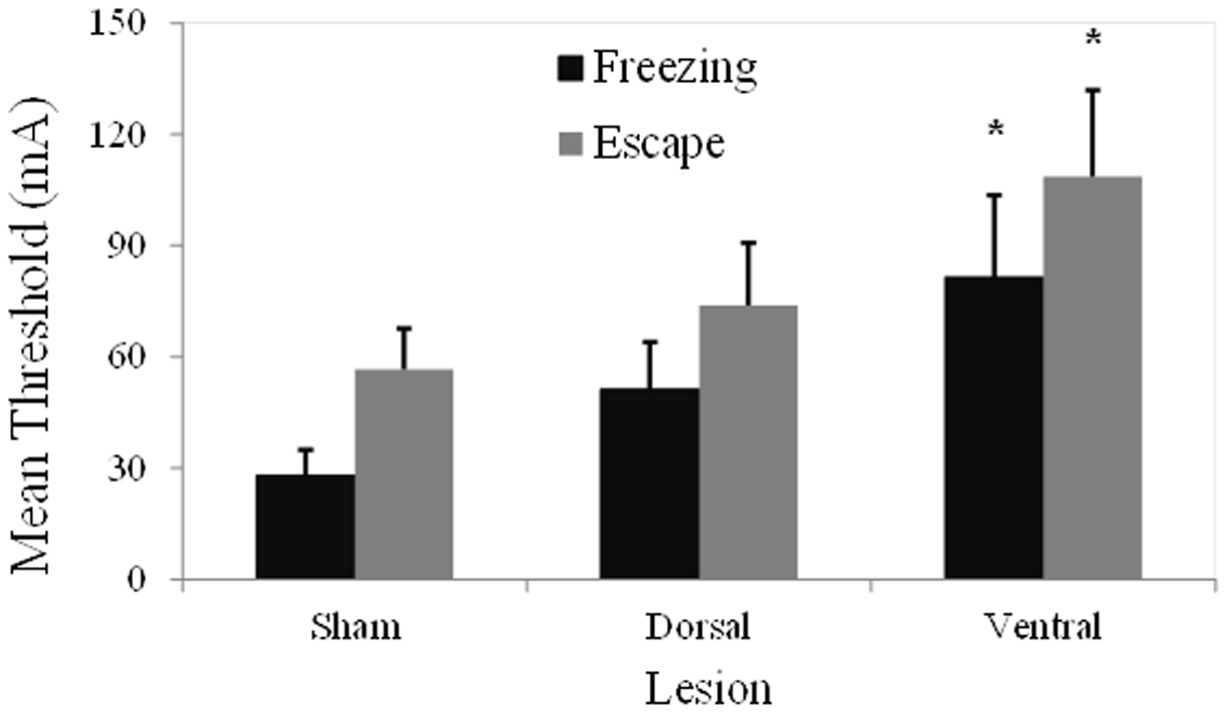
Mean (± SEM) freezing and escape thresholds induced by dPAG electrical stimulation in sham-, DH-, and VH-lesioned animals.

Fig. 2 presents the mean (± SEM) freezing and escape thresholds induced by electrical stimulation of the dPAG area. As previously reported [2], freezing and escape occurred in a stepwise fashion. At lower current intensities, freezing induced by electrical stimulation of the dPAG was characterized by the sudden cessation of all movements, except those necessary for respiration, accompanied by piloerection and exophthalmus. At higher current intensities, this freezing behavior was followed by vigorous running and jumping reactions. The escape response stopped as soon as electrical stimulation of the dPAG stopped. No differences in freezing (*t*_10_ = 0.01, *p*>0.99) and escape (*t*_10_ = 0.98, *p*>0.35) thresholds were observed between the two sham groups. Therefore, the data of these two groups were collapsed into a single group. The two-way ANOVA revealed no interaction between lesioning and aversive thresholds (*F*_2,24_ = 0.26, *p* = 0.76). Main effects of lesioning (*F*_2,24_ = 3.42, *p* = 0.05) and aversive threshold (*F*_1,24_ = 46.67, *p*<0.001) were found. *Post hoc* comparisons indicated that aversive freezing thresholds were consistently lower than escape thresholds (all *p*<0.05). Pairwise comparisons also revealed that VH-lesioned animals presented higher aversive dPAG electrical stimulation thresholds for freezing and escape responses compared with sham-lesioned animals (both *p*<0.05).

**Figure 3:**
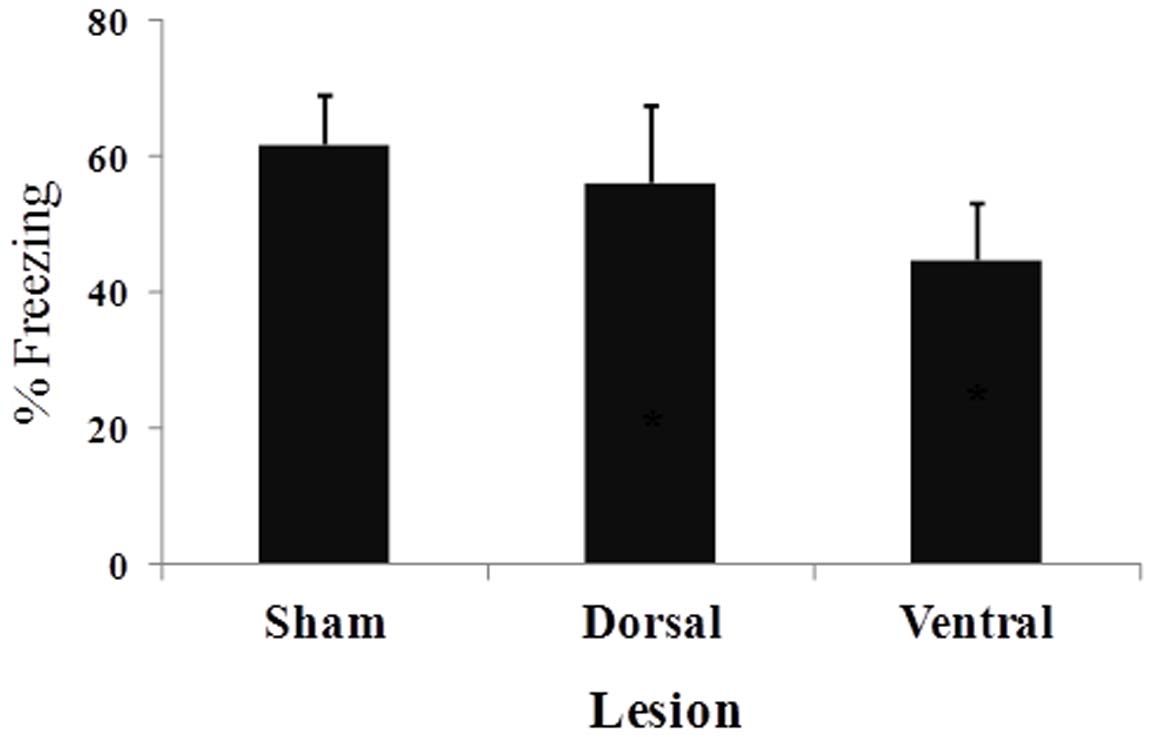
Mean (± SEM) percentage of freezing during the 8-min period after the cessation of dPAG stimulation applied at the escape threshold in sham-, DH-, and VH-lesioned animals.

Fig. 3 depicts the mean (± SEM) percentage of time that sham-, DH-, and VH-lesioned animals spent freezing after stimulation of the dPAG at the escape threshold. No difference in freezing behavior was found between the two sham groups (*t*_10_ = 1.45, *p*>0.17). Therefore, the data of these two groups were collapsed into a single group. The one-way ANOVA indicated no significant difference among the three groups (*F*_2,24_ = 0.4, *p*>0.66).

**Figure 4:**
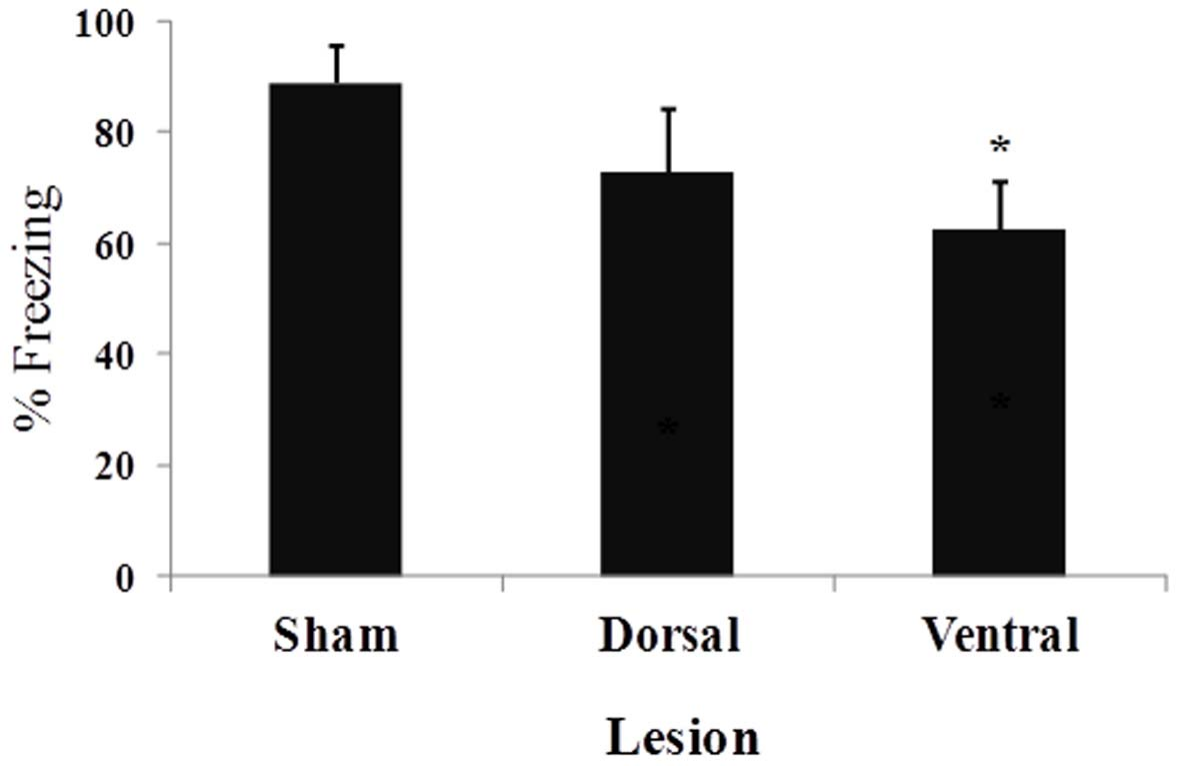
Mean percentage (± SEM) of freezing during the 2-min post-footshock period in sham-, DH-, and VH-lesioned animals.

**Figure 5:**
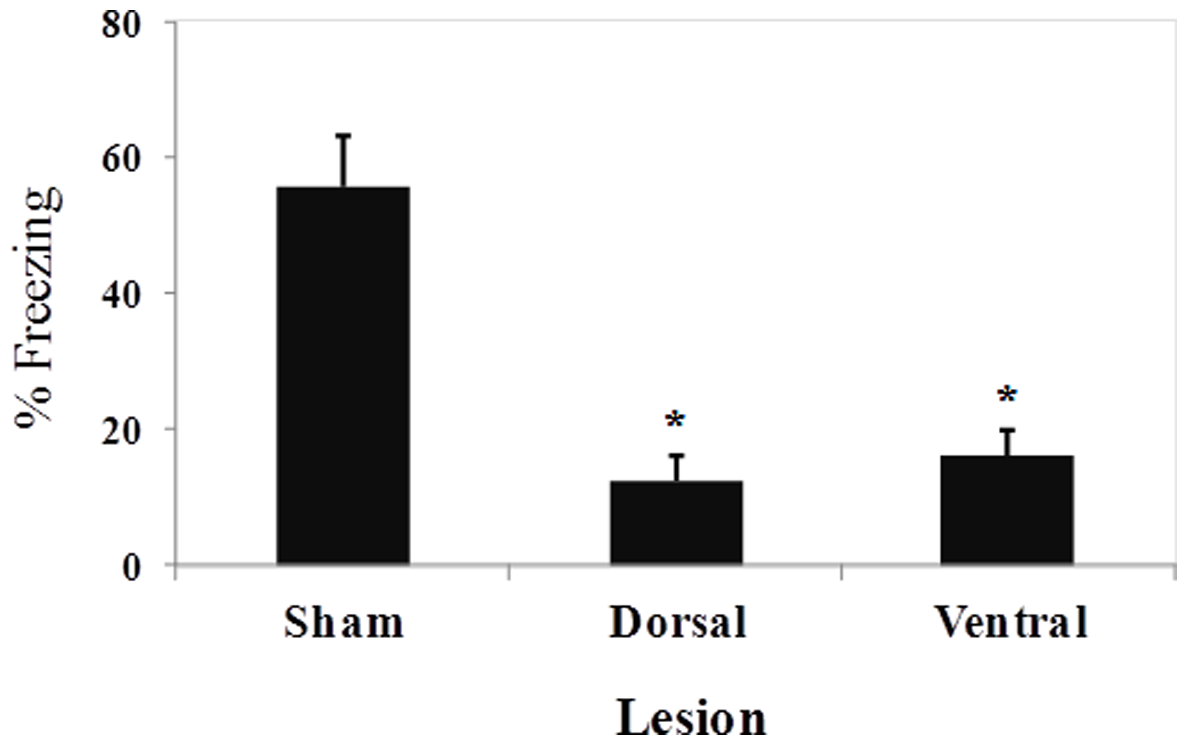
Mean percentage (± SEM) of conditioned freezing during the 8-min test session in sham-, DH-, and VH-lesioned animals.

Fig. 4 presents the mean (± SEM) percentage of time spent freezing during the 2 min period after the three footshocks that occurred during the contextual fear conditioning training session. No difference in freezing behavior was found between the two sham groups (*t*_10_ = 0.51, *p*>0.61). The one-way ANOVA indicated a significant difference among the three groups (*F*_2,24_ = 3.72, *p*<0.05). Pairwise comparisons indicated that VH lesions significantly reduced the duration of post-footshock freezing compared with sham-lesioned animals (*p*<0.05). Fig. 5 shows the mean (± SEM) percentage of time spent freezing during the 12 min test session that occurred 24 h after training. No difference in freezing behavior was found between the two sham groups (*t*_10_ = 0.35, *p*>0.72). The one-way ANOVA indicated a significant difference among the three groups (*F*_2,24_ = 14.54, *p*<0.001). Pairwise comparisons indicated that both DH and VH lesions disrupted contextual fear conditioning, lowering the amount of freezing behavior compared with sham-lesioned animals (both *p*<0.05).

## Discussion

The present study indicated that bilateral electrolytic lesions of the DH disrupted conditioned freezing 24 h after contextual fear training, whereas VH lesions reduced both defensive freezing behaviors immediately after footshock. These results are consistent with previous reports [6]–[10], [20] and corroborate the fact that the hippocampus is a complex brain structure involved in different aspects of contextual fear conditioning. Although still unclear are the underlying mechanisms involved in the participation of the DH in contextual fear conditioning [26], this hippocampal subregion appears to be critical for spatial processing, and the disruptive effect of DH lesions on contextual fear conditioning might be attributable to a detrimental effect on the encoding of contextual information during fear conditioning. Indeed, the DH receives highly processed, polymodal sensory information from the entorhinal and perirhinal cortices which, in turn, are the main recipients of different combinations of sensory information from unimodal and polymodal neocortical areas [27], [28]. The VH, in turn, has a distinct neuroanatomical profile that is closely associated with emotional responses. It receives direct projections from limbic structures, such as the hypothalamus, prefrontal cortex, and amygdala [29]–[31]. The fact that VH but not DH lesions disrupted freezing immediately after footshock might reflect the selective involvement of this subregion in defensive emotional reactions.

The differential roles that these two hippocampal areas might play in defensive behavior can also be suggested based on the dPAG electrical stimulation results. Bilateral electrolytic lesions of the VH but not DH produced an anti-aversive effect, in which they increased the threshold of the electrical current needed to elicit freezing or escape responses when applied to the dPAG. These results represent an important finding for the comprehension of the neural circuitry involved in defensive behavior triggered within the brainstem. Previous results suggested that aversive dPAG electrical stimulation activated immediate defensive responses through descending output projections to more caudal brainstem structures that are involved in the motor performance of these defensive responses, independent of any telencephalic structures, such as the amygdaloid complex [32]. The fact that VH-lesioned animals had a higher aversive dPAG electrical stimulation threshold to produce freezing and escape reactions than sham-lesioned animals indicates that these dPAG-dependent defensive reactions encompassed ascending projections that reached the VH. Consistent with this view, neuroanatomical studies revealed that the dPAG sends projections to midline and intralaminar thalamic nuclei and caudal diencephalic nuclei, such as posterior hypothalamic and supramammillary nuclei which, in turn, send projections throughout the hippocampal formation [33], [34].

Finally, the present results indicated that neither VH nor DH electrolytic lesions interfered with defensive freezing behavior observed after the cessation of dPAG electrical stimulation at the escape threshold. These results support the hypothesis that post-dPAG stimulation freezing and conditioned freezing in response to contextual cues previously associated with footshock might be related to distinct functional systems [5]. Conditioned freezing in response to contextual cues decreased after DH or VH lesions, whereas post-dPAG stimulation freezing behavior did not change after lesioning these same hippocampal areas. Moreover, defensive reactions triggered by dPAG electrical stimulation and post-dPAG stimulation freezing also recruit different neural structures, in which the former but not the latter was sensitive to VH lesions. Interestingly, previous results indicated that inactivation of the amygdaloid complex with electrolytic lesions or microinjections of muscimol produced a pattern of results that was opposite to the pattern observed with electrolytic VH lesions (i.e., it did not change the dPAG electrical stimulation necessary to induce freezing and escape responses but reduced dPAG post-stimulation freezing [32], [35]. Therefore, neural circuitry that involves the VH and amygdaloid complex appears to mediate different aspects of defensive behavior generated during and after dPAG electrical stimulation.

In conclusion, the present results indicate that the DH and VH are differentially involved in the neural circuitry associated with defensive behavior. Electrolytic lesions within these two areas were able to disrupt contextual fear conditioning, but only VH lesions disrupted freezing behavior immediately after footshock. Lesions exclusively within the VH increased the thresholds of aversive freezing and escape responses to dPAG electrical stimulation. Neither DH nor VH lesions disrupted post-dPAG stimulation freezing. The fact that the lesions were made with an electrolytic current is an important limitation of the present study because the effects on the dPAG electrical stimulation threshold might be attributable to the fibers that pass through the VH. Therefore, future studies that use selective chemical lesions may further clarify the participation of the VH in the modulation of dPAG defensive behavior.
